# Simplicity and Complexity in Combinatorial Optimization

**DOI:** 10.3390/e28020226

**Published:** 2026-02-15

**Authors:** Kamal Dingle, Marcus Hutter

**Affiliations:** 1 Department of Mathematics and Natural Sciences, Center for Applied Mathematics and Bioinformatics, Gulf University for Science and Technology, Hawally 32093, Kuwait; 2 Google DeepMind, London N1C 4DN, UK; 3 School of Computing, Australian National University, Canberra, ACT 2601, Australia

**Keywords:** combinatorial optimization, Kolmogorov complexity, algorithmic information theory, algorithmic sufficient statistics, symmetry, geometrical frustration

## Abstract

Many problems in physics and computer science can be framed in terms of combinatorial optimization. Due to this, it is interesting and important to study theoretical aspects of such optimization. Here, we study connections between Kolmogorov complexity, optima, and optimization. We argue that (1) optima and complexity are connected, with extrema being more likely to have low complexity (under certain circumstances); (2) optimization by sampling candidate solutions according to algorithmic probability may be an effective optimization method; and (3) coincidences in extrema to optimization problems are *a priori* more likely as compared to a purely random null model.

## 1. Introduction

Combinatorial optimization problems are common in physics and computer science, where a certain geometry or configuration is sought to maximize a given objective function [1,2]. Decades of extensive study have been given to the practical question of how to find optima in such settings, but it is also interesting to consider more abstract and theoretical questions regarding general properties or relations among optima (if any). Here, we consider some such questions, namely the connection between optima and extrema, and simplicity and complexity.

To motivate these questions, note that intuitively we might expect that optima and simplicity are related in nature since the laws of physics possess a lot of symmetries, and the energetically lowest state will respect these symmetries unless the symmetry is broken. For instance, the lowest energy state of, e.g., electrodynamics is the (very symmetric) empty space. This is similar for quantum electro dynamics (QED) but in the presence of particles the symmetry can be broken. Still, often the lowest energy state retains some of the symmetries, e.g., in crystals and where the objective function stems from basic physics and engineering principles [1]. This suggests there might be some general relation between optima and simplicity [3]. On the other hand, it is well-known that many difficult optimization problems involve high complexity objective functions, and the optimum is also highly complex; for instance, frustration in physics, e.g., magnetic materials like spin glasses, can exhibit very complex low(est) energy states.

Additionally, there are also several examples of optima that simultaneously optimize several different functions, that is, geometries/configurations that are simultaneously optimal, or near optimal, for different objective functions. Consider these motivating examples: (a) Suppose we have, argmaxf(x)=argmaxg(f(x)) for any monotone increasing function *g*, then different *g* will be optimized by the same *x*; (b) There exists a universally optimal arrangements of points on a sphere, which is optimal for a range of different energy functions [4]; (c) In studies of lattice proteins—a simplified biophysics model of protein folding—it has been observed that a subset of (simple and regular) lattice protein folds have the highest probability (i.e., have the most sequences assigned) [5], are the fastest folders [6], the fastest unfolders [7], the most thermally stable [5], and are highly robust to genetic mutations [8] (see also [9]); (d) Scale-free network architectures, with their regular patterns have been found to be highly robust [10], and also efficient [11] due to having small network diameters. Scale-free networks show the small-world property—indeed, ‘ultrasmall’ [11]: L∼loglogN where *L* is mean length between nodes. These networks are more efficient for small network diameters [11] and so the “resulting graph[s] includes, for free, an enormous homeostasis against random failure.” [10].

These examples hint at some potential general relations between simplicity, complexity, and optimization, and also potentially between optimizing one function and optimizing an apparently unrelated function. The theoretical framework we adopt in exploring these relations is *algorithmic information theory* [12,13,14], also known as Kolmogorov complexity theory. While there are many different ways to conceive of and measure “complexity” [15,16], Kolmogorov complexity is a natural and powerful approach which is well established in computer science and has a broad theoretical base of results. We examine various scenarios in which the combinatorial problem is either simple or complex, and in which cases the optimum will be simple or complex. Extending the investigation into simple and complex optima, we study connections between simple optima and how certain configurations may optimize different constraint functions, and relatedly, how employing sampling methods based on configuration complexity may yield effective optimization methods.

## 2. Background and Problem Set up

### 2.1. Theoretical Framework

Developed within theoretical computer science, *algorithmic information theory* [12,13,14] (AIT) sits at the intersection of computability theory and information theory. AIT is fundamentally based on estimating the information content, or complexity, of discrete objects or patterns, such as discrete sequences, or geometries. These information estimates can yield mathematical relations and bounds, which can be used for various scientific and mathematical predictions.

In AIT, information content is quantified by *Kolmogorov complexity*, K(x), which measures the complexity of an individual object *x* in terms of the amount of information required to describe or generate *x*. Another way to think about K(x) is via compression, where K(x) measures the size of the compressed version of *x* (assuming a perfect compressor). If *x* is a simple string e.g., containing repeating patterns like x=101010101010101010 then it is easy to compress, and hence K(x) will be small. Contrast this with a randomly generated bit string of length *n*, which is highly unlikely to contain any patterns, and hence cannot be compressed. In general, a sequence of length *n* is called “complex” or “random” if K(x)≈n bits, and “simple” if K(x)≪n.

More formally, the Kolmogorov complexity $K_{U}\left(x\right)$ of a string *x* with respect to a (prefix optimal) universal Turing machine [17] (UTM) *U*, is defined [12,13,14] as

(1) $$K_{U}\left(x\right)=\min_{p}\left\{|p|:U\left(p\right)=x\right\}$$

where *p* is a binary program for *U*, and |p| denotes the length of the (halting) program *p* in bits. Due to the invariance theorem [18] for any two optimal UTMs *U* and *V*, $|K_{U}\left(x\right)-K_{V}\left(x\right)|\leq c$ so that the complexity of *x* is independent of the choice of the machine, to within an additive constant *c*. Hence, we conventionally drop the subscript *U* in $K_{U}\left(x\right)$, and speak of ‘the’ Kolmogorov complexity K(x).

The quantity K(x) is formally uncomputable, meaning that there cannot exist a general algorithm that for any arbitrary string returns the value of K(x). This is related to the famous Halting Problem, and the logic of self-referential statements. In practice, K(x) is commonly approximated by standard data compression algorithms [18] (but see refs. [19,20,21] for other approaches). Note that Shannon information and Kolmogorov complexity are related [22], but differ fundamentally in that Shannon information quantifies the information or complexity of a random source, while Kolmogorov complexity quantifies the information of individual sequences or objects.

While AIT contains many abstract results, a large number of studies have shown that AIT and Kolmogorov complexity can be successfully applied in physics, including thermodynamics [23,24,25,26,27], entropy estimation [28,29], in addition to applications in engineering and statistics [18,30,31], and biology [32,33]. More details and technicalities can be found in standard AIT references [18,34,35,36].

### 2.2. Problem Description

We consider a general combinatorial optimization problem to take the form: find the optimal geometry/configuration/sequence $x^{*}$ where

(2) $$x^{*}=\arg\max_{x}f\left(x\right)$$

with x∈X, and f(x) is the objective function to maximize (there may be more than one optimal solution). The set X is assumed to be some finite set of discrete geometries, configurations, or sequences, such as binary strings of length *n* bits, graphs on *n* nodes, the set of self-avoiding walks of length *n* on a lattice, etc. The value of *n* is a ‘natural’ measure of the size of the problem. We assume that we can enumerate all possible elements within X.

Note that Kolmogorov complexity is distinct from computational complexity (which examines the amount of time or computational resources required for computation). We assume that the objective function f(x) is some computable function that can be evaluated e.g., on a computer. Being computable means that the function can be evaluated in finite time by mechanical means. Combinatorial optimization problems in physics and computer science often come in this form of Equation (2), just described [2].

## 3. Complexity of Optima, Sets, and Objective Functions

### 3.1. Bounding the Complexity of Optima

It is common practice in AIT to form bounds or estimates on the complexity of objects via bounding the descriptions of the objects. By “describe” or “specify”, we mean uniquely identifying an element from a set. We will bound the complexity of optima (or extrema) as follows: We can describe (or specify) any x∈X, given the value of *n*, by first generating the set X using *n*, then producing an ordering of the elements of X using the function *f* (i.e., ordering elements from most optimal to least), and then describing *x* exactly with its rank *r* in the ordering [37]. Thus, we can bound the Kolmogorov complexity of optima by implementing the following algorithm:

(i) Enumerate in order all elements $x_{i}\in X$
(ii) For each $x_{i}\in X$, evaluate $f(x_{i})$
(iii) List $x_{i}$ in descending order of optimality: $x_{1}^{*}$, $x_{2}^{*}$, …, $x_{\left|X\right|}^{*}$
(iv) Set $x^{*}=x_{1}^{*}$, print $x^{*}$ and halt

Any element in the list $x_{1}^{*}$, $x_{2}^{*}$, …, $x_{\left|X\right|}^{*}$ can be identified by its rank *r* in the list, where r=1 corresponds to the optimum $x^{*}$, r=2 indicates the second highest value for *f*, and r=|X| is assigned to the geometry with the lowest value of the objective function. Note that in keeping with the nomenclature of statistical physics we will use the words “geometry” or “configuration”, but this should not be read in the restrictive sense. Our results apply more generally to situations where merely “object” or “element” or “sequence”, etc., is more appropriate to the context.

If evaluating the objective function for different elements leads to the same function value, then this could lead to multiple elements with the same rank *r*. To avoid this and keep all ranks unique, we will assume that elements with the same value are also ranked according to the order they appear in the enumeration. For example, if $f\left(x_{45}\right)=f\left(x_{52}\right)$ and $x_{45}$ has rank *r*, then $x_{52}$ will have a larger rank because it appeared later in the original enumeration.

Assuming that the value of *n* is given, then the number of bits required to describe the rank *r* is K(r|n). The number of bits to describe the function *f* is K(f|n), and the number of bits to describe the full set of possible geometries/configurations is K(X|n). Because the rank *r*, the function *f*, and the set X can be used to describe $x_{r}^{*}$, then we can simply add the preceding together and bound the complexity $K(x_{r}^{*}|n)$ of any geometry $x_{r}^{*}$ by

(3) $$K\left(x_{r}^{*}|n\right)\leq K\left(X|n\right)+K\left(f|n\right)+K\left(r|n\right)+O\left(1\right)$$

From this bound, we see that the complexity K(X|n) of the set X as well as the complexity K(f|n) of the function *f* are important quantities. We will now examine these quantities in more detail.

### 3.2. Complexity of the Set

The set X may in general be low or high complexity, taking on any complexity value on a spectrum from zero to any positive integer value. Many optimization problems in physics and computer science take the form of looking for an optimal *x* over all possible *x* within some set, such as all possible graphs up to some size, all possible protein configurations, all possible branching patterns, all possible topologies with some condition. Perhaps surprisingly, the “set of all” objects of some kind typically has very low Kolmogorov complexity. For example, the set of all binary strings of length *n*, ${\left\{0,1\right\}}^{n}$, has complexity only

(4) $$K({\left\{0,1\right\}}^{n}|n)=O\left(1\right)$$

because it can be described by the program “Enumerate and print all binary strings of length *n*”. Contrast this with the complexity of a typical random element $x\in {\left\{0,1\right\}}^{n}$ which has complexity

(5) $$K\left(x\right)\approx n+O(\log_{2}\left(n\right))$$

far higher than O(1) bits.

Another example is *The Library of Babel* by Borges [38], which is a fictional library collection of all possible books of 410 pages. The information content of the *entire* library is almost zero, because it can be generated via a trivial algorithm enumerating all possible letter combinations. In stark contrast, almost all books in the library have high information content, because describing one precisely would require hundreds of pages of text (in a typical case). Indeed, the countably infinite set of *all* binary strings {0, 1, 00, 01, …} has very low information content, because it can be generated by a fixed length program, and hence has complexity O(1) bits. Similarly, the set of all graphs on *n* nodes has very low complexity, at most ≈$\log_{2}\left(n\right)$ bits. This discussion shows that in many scenarios of relevance to computer science or physics, the set X maybe be very simple (low complexity).

Despite the preceding, a set can be complex if many bits are required to specify it precisely. For example, if X is made from randomly selecting $N\ll 2^{n}$ strings out of all binary strings of length *n*, then in this case

(6) $$K\left(X|n\right)\approx N\cdot n\gg K({\left\{0,1\right\}}^{n}|n)=O\left(1\right)$$

because the only way to precisely generate this set X is by individually specifying each element, each of which has complexity ≈n bits (with high probability, as in Equation (5)). Similarly, a set of data taken from the internet, such as a set of films or articles, will typically have high complexity, being constituted of high-information content images and text. Within physics, a concrete example would be a physical system with *f* being the energy which is typically a simple function, but with a complex boundary, e.g., waves in some irregularly shaped pond. Having said that, you can always integrate the complexity of X into *f* by enlarging the domain of *f* to some very simple domain, and then setting f=∞ outside X.

### 3.3. Complexity of the Objective Function

An objective function f(x) may in general be low or high complexity, again on a spectrum of possible complexity values. Low complexity functions will be those which can be described with short programs, or via brief mathematical rules. Examples of common simple functions are (low order) polynomials, trigonometric functions, and the Hamiltonian (energy) of a system. These all are classed as simple O(1) complexity functions, because they can be computed via small fixed-sized algorithms.

A function is high complexity if specifying it requires many bits, typically via a long and detailed program. An example of a complex function is

(7) $$f\left(x\right)=\min_{x\in X}H\left(x,z\right)$$

where *H* is the Hamming distance between binary strings *x* and *z*, with  *z* a random string of length *n*. In this case, K(f|n)≈n bits, which may be arbitrarily large.

### 3.4. Constraints

Another instance where the objective function can effectively have high complexity is if there are high-complexity boundary conditions or constraints which must be satisfied. For example, in non-trivial linear programming problems, the number of constraints can be large, and each constraint is specified by some essentially random values (each with high information content). While this is an important practical consideration for many optimization scenarios, we will not specifically investigate these types of constraints here in this work (but formally this is equivalent to restricting/describing the domain X).

## 4. Simplicity and Complexity in Optima and Extrema

Having considered different possible Kolmogorov complexity scenarios of objective function and sets, and derived an upper bound on the related combinatorial optimization problems, we will now consider four types of problem in terms of the simplicity or complexity of optima. Each of these four types has a different relation of simplicity to complexity: Simple (low-complexity) optimization problems with simple (low-complexity) solutions; simple optimization problems with (apparently) high complexity solutions; high complexity problems with high complexity solutions; and high complexity problems with simple solutions.

### 4.1. Simplicity from Simplicity

Above, we argued that many optimization problems relevant to physics and computer science can have simple sets with simple objective functions. In these cases, assuming that *n* (which quantifies the size of the system) is given, we have that K(X|n)=O(1) and K(f|n)=O(1) are small, fixed-sized O(1) complexity objects. Further, highly optimal structures *x* will have rank values r≈1 (and r=1 for a unique optimum) and hence K(r|n)≈0. Therefore, the right hand side of Equation (3) will be small, and we can write

(8) $$K\left(x_{r}^{*}|n\right)\leq K\left(X|n\right)+K\left(f|n\right)+K\left(r|n\right)+O\left(1\right)\;\;\;⟹\;\;\;K\left(x_{r}^{*}|n\right)=O\left(1\right)$$

so that any $x_{r}^{*}$ with r≈1 must have low Kolmogorov complexity (i.e., “simple”), and $x^{*}$ must have very low, O(1) complexity. Further, if there are multiple global optima, and we are only concerned about one of these but not which one, then we can still choose r=1. If we are concerned about all global optima, the complexity of the set of all global optima is also still O(1). Only if we are concerned about a specific optimum, then by definition there is a secondary criterion, and if this is simple, then its complexity is still O(1).

Our argument shows formally how simplicity in optima can arise from simplicity of the optimization problem. It is true that we have assumed that *n* is given here, and so it is in principle possible that the complexity of *n* itself will lead to a higher complexity of $x_{r}^{*}$. However, it is unlikely that the effect is substantial because firstly the value *n* contains at most $O(\log_{2}\left(n\right))$ bits, which is much smaller than typical complexity values of objects in the set. Secondly, there are many ‘special’ and highly compressible values of *n*, for which $K\left(n\right)\ll \log_{2}\left(n\right)$ and so there is even less of a possibility that the complexity of *n* itself strongly influences the complexity of $x_{r}^{*}$. Taking a scientific example, a kilogram of water may contain *n* = 33, 428, 104, 385, 201, 608, 245, 856, 294 water molecules with K(n)≈90 bits, but if instead we take $n=3.3428\times 10^{25}$, then the complexity is reduced substantially to K(n)≈20, and yet will likely not change the optimization problem. Nonetheless, it remains a possibility.

We have focused on optimal solutions, due to the inherent interest, but the derivations apply also to extrema more generally, including configurations which minimize or extremize some function rather than maximize. That is, worst possible solutions as well as best possible solutions may be simple. To see why, note that K(r|n)=O(1) also holds for poorly ranked configurations with r≈|X|. For example, if there are $2^{n}$ possible geometries, then the last rank will be $r=2^{n}$, and hence $K\left(r|n\right)=K\left(2^{n}|n\right)=O\left(1\right)$. Therefore, the *least* optimal geometry must also have low Kolmogorov complexity (assuming a simple set and function). In general, any geometries with extreme values with respect to the objective function will be low complexity, and so extrema, not just optima, must be low complexity.

**Example** **1.** *We give an example illustrating how simplicity can come from simplicity in combinatorial optimization. Define $X={\left\{0,1\right\}}^{n}$, and let the objective function be*

(9) $$\begin{array}{c} f\left(x\right)=\sum_{i=1}^{n}x_{i} \end{array}$$

*with $x=(x_{1},x_{2},\ldots ,x_{n})\in X$, i.e., f(x) counts the number of 1’s in the string. The set X has complexity K(X|n)=O(1), and K(f|n)=O(1). It follows then from Equation (8) that the optimal string $x^{⋆}$ with r=1 must have $K\left(x^{⋆}|n\right)=O\left(1\right)$ and hence be very simple. In this somewhat trivial example, we can confirm this prediction by noting that the optimal solution is $x^{⋆}=\left(1,1,1,\ldots ,1,1\right)$ with n 1’s, and we know that K(111…11|n)=O(1). It is also noteworthy that the worst solution (0,0,0,...,0,0) is an extrema, and is also very simple.*

 

**Example** **2.** *We give another example, this one less trivial and coming from biophysics. RNA molecules fold into structures which are important for their function in the cell. The secondary structure of an RNA molecule refers to the bonding pattern of chemical bases, and it is common in bioinformatics to represent the secondary structure in dot-bracket form, such as (((...))).... which can be explained via this diagram below: 
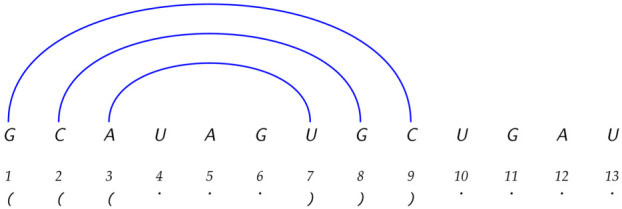
 This means that base 1 is chemically bonded to base 9, and base 2 is bonded to base 8, and base 3 is bonded to base 7. The other bases (numbered 4, 5, 6, 10, 11, 12, 13) are not chemically bonded to any other base. Different underlying sequences give rise to different bonding patterns, and these bonding patterns define the RNA secondary structures. For a sequence of length L bases, there are around $1.76^{L}$ [39] different possible (valid) secondary structures. Underlying these structures are $4^{L}$ possible sequences of length L made up of the letters A, T(U), C, and G. The set of all RNA sequences can be obtained by merely enumerating all possible sequences in order, which is a simple O(1) complexity set, assuming we are given L. Also, the mapping [40] which generates a given structure from a sequence is O(1) [31] for RNA folding (structures are adopted based on free energy values). Therefore, the set of all structures of length L is O(1) complexity, given L.*

*We can set up an optimization problem as follows: enumerate all secondary structures x and for each, let f(x) be the number of RNA sequences (from the $4^{L}$ total) which adopt that structure x as a minimum free energy fold. This is the score for the structure, where higher scores are more optimal. Sort secondary structures by these scores, highest to lowest. According to our arguments above, the highest scoring (optimal) structure must have low Kolmogorov complexity. In fact, it is known [31] that the optimal structure is the trivial structure with no bonds at all, which is extremely simple.*

### 4.2. Complexity from Simplicity

We have proven that extrema and optimal solutions must have low Kolmogorov complexity under certain conditions, but this is not necessarily the same as simple, symmetric, or regular in the usual senses of the words. It is possible, in principle, that these solutions appear complex or pseudo-random. Kolmogorov complexity defines simplicity in terms of program length, and all intuitively simple (i.e., symmetric, repeating, regular) patterns must have low Kolmogorov complexity. But the opposite does not hold; there may be irregular patterns that have low complexity. For example fractals are low complexity, but not intuitively ‘simple’. Hence, for some simple optimization problem with low Kolmogorov complexity, it may appear to be complex. In this case, we might have a form of complexity arising from a simple (low complexity) problem. Having said that, we stress that this ‘complexity from simplicity’ is only apparent, and not true high (Kolmogorov) complexity.

**Example** **3.** *As an example of (apparent) complexity from simplicity in optimization, consider the example,*

(10) $$f\left(x\right)=\min_{x\in X}H\left(x,z\right)$$

*where H is the Hamming distance between $x\in {\left\{0,1\right\}}^{n}=X$ and z, and z some pseudo random binary string of the same length as x, such as the first n digits of π=3.141… when given in binary form. The number π is a textbook example of a pattern which appears complex, but in fact follows a deterministic pattern, hence having low Kolmogorov complexity.*

Having highlighted the potential distinction between low Kolmogorov complexity objects and intuitively simple objects, it is interesting to consider how often such low complexity but irregular patterns might arise in physics and the natural world. On the one hand, Wolfram [41,42] has argued for the ubiquity of complexity in natural systems, even arising from simple deterministic rules. Similarly, the ubiquity of chaotic systems in nature, such as the weather, would support this view. On the other hand, we might expect in many natural systems that near optimal solutions will in fact be simple, or regular, or symmetric. This is due to Vitanyi [30,43], who has given an argument for why common patterns in natural science are unlikely to be pseudo-random, pointing out that common physics-based objective functions are unlikely to contain universal Turing machines which can compute arbitrary algorithms and generate pseudo-random patterns.

Hence, even though we have not proved that optima must be simple in an intuitive sense, in many settings relevant in physics low Kolmogorov complexity geometries may be more likely to be intuitively simple as compared to typical configurations (under certain conditions).

### 4.3. Complexity from Complexity

If the set or objective function is complex, then the optimum can be complex. That is, if the RHS of Equation (3) is large, it is possible that the LHS is also large (complex). This scenario is relevant to many optimization problems in computer science and physics (and indeed more so in other areas of science, such as biology and ecology). This would be complexity arising from complexity.

To assess whether the optimum is indeed high complexity, we can examine the conditional complexity $K(f,X|x^{*})$, which is the minimum number of bits required to generate the function *f* and the set X, given access to $x^{*}$ (defined in Equation (2)). Assume here that $x^{*}$ is the unique optimum solution. Intuitively, if this conditional complexity is low, then the optimum solution $x^{*}$ contains a lot of information about the function *f* and set X, hence must be complex itself. More formally, we examine the ratio

(11) $$\alpha \equiv \frac{K(f,X|x^{*})}{K(f,X)}$$

where we assume that K(f,X) is high complexity, i.e., not O(1) complexity, so that it is unbounded for large *n*. If α≪1 and K(f,X) is large, then the optimum $x^{*}$ must be complex, due to the following argument:

(12) $$\begin{array}{cc} K(f,X) & \leq K\left(x^{*}\right)+K\left(f,X|x^{*}\right)+O\left(1\right) \end{array}$$

(13) $$\begin{array}{cc} \Rightarrow \frac{K(f,X)}{K(f,X)} & \leq \frac{K(x^{*})}{K(f,X)}+\frac{K(f,X|x^{*})}{K(f,X)}+O\left(\frac{1}{K(f,X)}\right) \end{array}$$

(14) $$\begin{array}{cc} \Rightarrow 1 & ≲\frac{K(x^{*})}{K(f,X)}+\alpha \end{array}$$

Because α≪1 then

(15) $$1-\alpha \approx 1≲\frac{K(x^{*})}{K(f,X)}\Rightarrow K\left(f,X\right)≲K\left(x^{*}\right)$$

so that the optimum $x^{*}$ is complex. In other words, if knowing the optimum helps substantially in describing the function and set, then it must be complex.

If instead we have α≈1, then intuitively $x^{*}$ must be simple, as established by the following argument: it is known [18] that for Kolmogorov complexity, to within logarithmic accuracy, we have

(16) $$\begin{array}{cc} \; & K(x,y)\approx K(x|y)+K(y) \end{array}$$

(17) $$\begin{array}{cc} \; & K(y,x)\approx K(y|x)+K(x) \end{array}$$

(18) $$\begin{array}{cc} \; & K(x,y)\approx K(y,x) \end{array}$$

for any binary strings *x* and *y*. Using these relations, we can write

(19) $$\begin{array}{cc} \; & K\left(x^{*},f,X\right)\approx K\left(x^{*}\right)+K\left(f,X|x^{*}\right) \end{array}$$

(20) $$\begin{array}{cc} \; & K\left(x^{*},f,X\right)\approx K\left(f,X\right)+K\left(x^{*}|f,X\right) \end{array}$$

(21) $$\begin{array}{cc} \; & \Rightarrow K\left(x^{*}\right)+K\left(f,X|x^{*}\right)\approx K\left(f,X\right)+K\left(x^{*}|f,X\right) \end{array}$$

and because α≈1, and $K\left(x^{*}|f,X\right)=O\left(1\right)$, we have

(22) $$\begin{array}{cc} \; & K\left(x^{*}\right)+K\left(f,X\right)\approx K\left(f,X\right)+O\left(1\right) \end{array}$$

(23) $$\begin{array}{cc} \; & \Rightarrow K\left(x^{*}\right)\approx O\left(1\right) \end{array}$$

so that the optimum $x^{*}$ will be low complexity (to within logarithmic accuracy). We return to the case of simple optima for complex problems later in this work.

**Example** **4.** *As an example of a complex optimum arising from a complex problem, consider the optimization problem*

(24) $$f\left(x\right)=\min_{x}H\left(x,z\right)$$

where H is the Hamming distance between $x\in {\left\{0,1\right\}}^{n}=X$ and z, with z a random binary string of same length as x. The optimum x solution will of course be $x^{*}=z$ so that the complexities $K\left(x^{*}\right)=K\left(z\right)\approx n$ are equal. This shows complexity arising from complexity. Note also that the extrema corresponding to the worst performing string, namely the binary string with all of the bits of z flipped, also has high complexity. Note also that in the case of the optimum we have

(25) $$\alpha =\frac{K(f,X|x^{*})}{K(f,X)}=\frac{K(z|x^{*})}{K(z)}\approx \frac{O(1)}{n}\ll 1$$

because $x^{*}$ contains most of the information of the optimization problem.

 

**Example** **5.** *A notable example of a complex function arises in the well-known assignment problem from combinatorial optimization [2]: If there are n people and n jobs, and a matrix $C_{ij}$ giving the affinity of person i for job j, the problem is to find the assignment of the jobs to the people which maximizes the total affinity. The space of configurations/sequences is the space of the n! permutations of the n people. Here, the objective function f(x) is in general not simple, because the Kolmogorov complexity of the matrix $C_{ij}$ will typically be $O(n^{2})$ for a random matrix. The Kolmogorov complexity of a typical permutation, and a typical optimal solution, is (using Stirling’s approximation)*

(26) $$\begin{array}{c} K\left(x\right)\approx \log_{2}\left(n!\right)+O\left(\log_{2}\log_{2}\left(n!\right)\right)∼\log_{2}\left[{\left(n/e\right)}^{n}\right]∼n\log_{2}\left(n\right) \end{array}$$

*which is complex.*

 

**Example** **6.** *Consider the example of the Ising model (external magnetic field is zero; example suggested by an anonymous reviewer) from statistical physics*

(27) $$f\left(x\right)=-\sum_{\left(i,j\right)}J_{ij}x_{i}x_{j}$$

*and $x_{i}$ is a binary value, either 0 or 1, with i=1,2,…,n. This classic problem in combinatorial optimization is to find the ground state $x=(x_{1},x_{2},\ldots ,x_{n})$, which minimizes the energy of the system. If the couplings are random variables $J_{ij}=\pm 1$, then the objective function is complex because each of these values requires 1 bit to specify whether +1 or −1. The optimum solution (if unique) is also complex, as is well known in statistical physics and computer science. As a side comment, if we were to replace random $C_{ij}$ or random $J_{ij}$ by pseudo-random, then the functions and solutions would become low complexity.*

 

**Example** **7.** *We could have a simple function f(x), but at the same time a complex set, e.g., the set is made up of a random subset of ${\left\{0,1\right\}}^{n}$. In this case, the optimum solution could still be complex, even though the function itself is simple.*

### 4.4. Simplicity from Complexity

Finally, we turn to the case where simplicity can arise from complexity. This is perhaps the most intriguing case. Having said that, the notion that simple laws can arise from complex phenomena is well established in e.g., statistical physics [44].

Looking to Equation (3), the fact that it is an upper bound allows for both a large RHS and small LHS, i.e., a complex function and or set, and yet a simple optimum solution. As mentioned above in relation to Equation (11), if the conditional complexity ratio is close to 1, then we can expect a low complexity optimum. But does this simplicity from complexity occur in any combinatorial systems? We give examples now.

**Example** **8.** Returning to the Ising model in Equation (27), if instead we impose that $J_{ij}\in \left\{1,2,3,\ldots ,M\right\}$ for some finite M, then we have a complex objective function. The complexity arises due to specifying each of the positive $J_{ij}$ values. Despite this complexity, the solution which maximizes the sum is simple, because the optimum is $x^{*}=\left(1,1,1,\ldots ,1,1,1\right)$. This simplicity follows from the fact that because all $J_{ij}>0$ the details of the specific positive values are irrelevant to identifying the optimal solution, and yet those details are part of the specification (hence complexity) of the function. It is easy to conceive of other cases where the function contains many details and so has high complexity, while at the same time these details are largely irrelevant to the optimum. In these cases, we will have simplicity arising from complexity.

To see another way in which simple optima can arise from complex objective functions, we explore this question from a different perspective. Within AIT, the notion of *algorithmic probability* [12,18,45,46] connects complexity and probability in a direct and profound way. Mathematically, it states that

(28) $$P_{U}\left(x\right)=2^{-K(x)+O(1)}$$

where $P_{U}$ is the probability that a random program run on a prefix universal Turing machine prints output *x*, and halts. The equality shows that simple, low complexity outputs are exponentially more likely than complex outputs. Thus, probability and complexity are two sides of a coin.

Algorithmic probability is not easy to apply directly to real-world problems, at least due to the uncomputability of K(x) but also due to the assumed presence of Turing machines. With these issues in mind, a weaker but more directly applicable bound was presented and tested in ref. [31]. The bound has the form

(29) $$P\left(x\right)\leq 2^{-a\tilde{K}\left(x\right)-b}$$

and is known as the *simplicity bias* bound. This bound applies to real-world computable input-output maps. Here, $\tilde{K}\left(x\right)$ denotes some computable complexity measure, such as lossless compression-based measures. The constants a>0 and *b* are independent of *x*. The motivation for including the constant *a* in the bound is that real-world complexity measures may not scale with Kolmogorov complexity. For example, a random binary string of length *n* has K(x)≈n, while some specific real-world complexity measure may scale like, say, 1.8n. Hence *a* can be adjusted to rescale the complexity appropriately. Similarly, the presence of the constant *b* is also included to adjust the complexity measure for appropriate scaling. For example, some specific real-world complexity measure may assign all objects a minimum complexity of 5000 bits. Hence, the values of *a* and *b* allow for re-scaling the complexity values. Having briefly discussed these, we note that the values of *a* and *b* are not relevant to our present theoretical work here, and details of their estimation and meaning are given in refs. [31].

The upper bound Equation (29) shows that if the probability distribution is nonuniform, it must be that the highest probability outputs are simple. It is not possible for complex outputs to have high probability. This bound has so far been applied to a wide variety of systems, including molecular shapes such as natural protein quaternary structures [33], RNA secondary structures [31,33], polyomino self-assembled 2D tile shapes [33,47], output patterns in machine learning [48,49,50], genetic programming [51], and natural [52] and dynamical system [53,54] time series data. Now, let us see how this bound could apply to an optimization problem, and yield simple optima from complex problems.

**Example** **9.** *We return to the RNA structure example from biophysics, described above. Suppose we have a set of RNA structures, X. We also have a set of RNA sequences, S, and a mapping function g:S→X. The function g is many-to-one, so that a given structure x may have many sequences s which map to it, and |X|≪|S|, and K(g)=O(1) [31]. The function g(s)=x maps sequences to structures by searching for the minimum free energy structure x∈X for the given sequence s, hence the mapping g can be viewed as combinatorial optimization. The complexity of the optimization problem depends primarily on K(s), and analogously to above we have*

(30) K(x)≤K(g)+K(s)+K(X|s)+O(1)=K(s)+O(1)

*because x and X can be generated by g and the sequence s, and an O(1) program. Now, consider the following algorithm:*

(i) *Enumerate in order all sequences s∈S*
(ii) *For each s∈S, evaluate g(s) to find the corresponding x*
(iii) *Record the frequency ϕ(x) with which each structure x appears, where $\sum_{x}\phi \left(x\right)=\left|S\right|$*
(iv) *Define $P_{\phi }\left(x\right):=\phi \left(x\right)/\left|S\right|$ as the probability of observing structure x on random choice of s*

*For RNA structures, it is known [31,33] that the simplicity bias relation of Equation (29) applies for a range of structure complexity values. Hence, $P_{\phi }\left(x\right)≲2^{-aK(x)}$, and there are (many) simple structures with high probability $P_{\phi }\left(x\right)$. Supposing x has high probability $P_{\phi }\left(x\right)$, then it follows that the preimage $g^{-1}\left(x\right)\subseteq S$ contains many random and complex sequences. By definition, each of these random and complex sequences mapped to a simple x via free energy minimization, hence these complex sequences had a simple optimum. In other words, there were many complex optimization problems with simple optima. It is easy to see that very similar arguments would yield examples of simple optima in related optimization problems, such as lattice proteins [5].*

## 5. Optimization by Algorithmic Probability Sampling

In the preceding sections, we have seen how simple optima can occur from simple optimization problems, but also from complex problems. Given the potential ubiquity of simple optima, it is interesting to consider if there is a way to exploit this to search for optima in combinatorial optimization problems.

Let us suppose we will attempt to solve an optimization problem by random sampling from X, where as before the size of the system is parameterized by *n*. Let us consider the expected waiting time to find the optimum (we will assume there is a unique optimum here), that is, the expected number of random samples to find the optimum. From the geometric distribution, we know that the expected waiting time to first observe some outcome which has probability *q* is 1/*q*. Therefore, if we sample all elements of X uniformly randomly, then q=1/|X|. For simplicity, we will assume that $\left|X\right|=2^{n}$, which would be the case if X is all binary strings of length *n*. Under these assumptions, the expected waiting time to find the optimum (assuming unique) is

(31) $$1/q=\left|X\right|=2^{n}$$

which is exponential in *n*. Hence, this is not an efficient manner to find the optimum. If instead we randomly sample elements of X according to their algorithmic probability, then the probability *q* of choosing the optimum is

(32) $$q=2^{-K\left(x^{*}|n\right)+O\left(1\right)}$$

so that the expected waiting time is

(33) $$1/q=2^{K\left(x^{*}|n\right)+O\left(1\right)}\leq 2^{n+O(1)}$$

because

(34) $$K\left(x^{*}|n\right)\leq n+O\left(1\right)$$

If you know the function and set is simple, then we expect the optimum to be simple (or there exists a simple optimum), then

(35) $$K\left(x^{*}|n\right)\ll n\;\;\;⟹\;\;\;1/q=2^{K\left(x^{*}|n\right)+O\left(1\right)}\ll 2^{n}$$

which means that the expected waiting time to find the optimum by sampling is exponentially faster than by uniform sampling, or much faster, even super-exponentially, e.g., if $K\left(x^{*}|n\right)=O\left(1\right)$. Interestingly, this suggests that sampling according to algorithmic probability is a good way to find the optimum even if you do not know the complexity of the optimum: if the complexity is high the number of samples you will need on average is

(36) $$K\left(x^{*}|n\right)\approx n+O\left(1\right)\;\;\;⟹\;\;\;1/q=2^{K\left(x^{*}|n\right)+O\left(1\right)}\approx 2^{n+O(1)}$$

Therefore, in the case of a complex optimum, the expected waiting time in terms of the number of samples is not very different as compared to from uniformly sampling. Summarizing, this means that sampling according to algorithmic probability will find the optimum much faster if it is simple, and not much slower if it is complex. Hence, it appears that sampling by algorithmic probability is an effective way to search for an optimum of optimization problems of varying complexity (note that we have ignored here the computational resources required to generate samples).

Whether this method is practically useful remains to be seen in future work. There are at least two problems to consider: (1) Kolmogorov complexity is uncomputable; and (2) that if complexity approximations are used, then computing the complexities may be time consuming, which adds to the computational complexity, and may be inaccurate. Nonetheless, there are known cases when sampling can be faster than optimization algorithms [55], and when random sampling itself largely explains generalization in machine-learning problems [56] (with further tuning of parameters via SGD improving, but not fully accounting for, generalization success). These cases shed light on the potential value and impact of sampling as a means of optimization.

This optimization method we proposed (i.e., sampling by algorithmic probability) is closely related to *Levin Search* [18], a method invented by Leonid Levin for inversion problems. The incomputable sampling process from $P_{U}\left(x\right)$ above is replaced by sampling from the computable Speed prior S(x) [57], which includes a penalty on the run-time of programs *p*. Some example applications of sampling from $P_{U}$ are (1) to show that average-case complexity equals worst-case complexity ([18], Thm.4.4.1) and (2) for training large language models (LLMs) [58]. If you replace sampling from Solomonoff’s $P_{U}$ by sampling from the Speed prior *S*, then you get a stochastic version of Levin search compared to Levin search which systematically evaluates all programs in increasing Kt-complexity, where $S\left(x\right)\approx 2^{-Kt(x)}$. Classical Levin search is applied to inversion problem, while we are studying optimization problems here. Unlike in search, a difficulty in optimization is that we may stumble upon the maximum quickly, but never know whether there is not a better solution. But such any-time algorithms are typical in AI, and Levin search has been extended to Levin Tree search with great practical success [59]. Hence, the proposed method of optimization by sampling is related to earlier work but differs in three ways: (1) optimization instead of inversion (as in Levin search); (2) using $P_{U}$ instead of *S*; and (3) sampling instead of enumeration.

## 6. Coincidences of Extrema

In the Introduction, we saw some examples of optimal geometries that simultaneously have high or optimal values for different objective functions, for example protein folds and network architectures. We now consider whether these are rare exceptional cases, or instead instances of a more general feature of combinatorial optimization.

### 6.1. Null Expectation for Simultaneously Optimizing Multiple Objective Functions

Consider a finite and simple set X as above, K(X|n)=O(1), and two different objective functions $f_{1}$ and $f_{2}$, with respective optima $x_{f_{1}}^{*},x_{f_{2}}^{*}\in X$. If these two functions are random functions, such that their values are random real variables (e.g., sampled from Gaussian distributions), then the null model prediction for the probability that the two optimal geometries coincide is

(37) $$P\left(x_{f_{1}}^{*}=x_{f_{2}}^{*}\right)=\frac{1}{\left|X\right|}$$

assuming that all function values are distinct. This probability is typically exponentially small, because |X| is typically exponential in *n*, e.g., $\left|X\right|=2^{n}$ if X consists of all binary strings. Hence, under the random function null model it is highly unlikely that the optimum geometry for one function coincides with that of another function.

The situation is very different for optima from simple functions: if $f_{1}$ and $f_{2}$ are simple, having O(1) complexity, then both $x_{f_{1}}^{*}$ and $x_{f_{2}}^{*}$ must be simple by Equation (8). Because there are exponentially few simple geometries, we know that both $x_{f_{1}}^{*}$ and $x_{f_{2}}^{*}$ must come from this exponentially small set. Hence, it is *a priori* far more likely that $x_{f_{1}}^{*}$ and $x_{f_{2}}^{*}$ coincide.

More formally, let $X_{O(1)}\subset X$ be such that all elements are simple with O(1) complexity. In this scenario, the null probability expectation that the two optima coincide is

(38) $$\left|X_{O(1)}\right|=O\left(1\right)\ll |X|\Rightarrow P\left(x_{f_{1}}^{*}=x_{f_{2}}^{*}\right)=\frac{1}{|X_{O(1)}|}\gg \frac{1}{\left|X\right|}$$

due to the fact that there are only O(1) simple geometries. While O(1) is not a precise quantitative value, nonetheless we understand that this probability is much higher than the exponentially small value of 1/|X|. The value $1/|X_{O(1)}|$ may still be low, but it is much higher than expected by random functions.

The preceding is an *a priori* argument, or expected null model for simple optima. It does not mean that all simple optima will be optima for all simple functions. Indeed, if we chose $f_{2}\left(x\right)=-f_{1}\left(x\right)$ then the optimum for $f_{1}$ will be the *worst* performing geometry for $f_{2}$. As discussed above, extrema must be simple, but not necessarily optimal.

Another reason why optima may not coincide is due the fact that while optima (under the described conditions) must be simple, this does not mean that all simple geometries must be optima. Some simple geometries may have low or medium values with respect to the objective function. This is related to the occurrence of low complexity, low probability patterns in studies of simplicity bias [60].

### 6.2. Coincidence of near Optima

The above argument can be extended beyond exactly coinciding optima, to geometries which are close to being optimal, i.e., with rank r≈1. We can ask, given we have an optimal solution $x_{f_{1}}^{*}$ for $f_{1}$, and we have a set $X_{f_{2}}^{r}\subset X$ consisting of the *r* most optimal geometries in X ranked according to $f_{2}$, what is the probability that $x_{f_{1}}^{*}\in X_{f_{2}}^{r}$? This is an interesting question, because if $x_{f_{1}}^{*}\in X_{f_{2}}^{r}$ then it means that the optimal geometry for one objective function $f_{1}$ is also close to optimal for another apparently unrelated objective function $f_{2}$. To proceed, if $f_{1}$ and $f_{2}$ are random functions, then we expect

(39) $$P\left(x_{f_{1}}^{*}\in X_{f_{2}}^{r}\right)=\frac{r}{\left|X\right|}$$

which is very small for highly optimal geometries with r≈1 because r≪|X|. Contrast this with the case where functions are simple, then the null model prediction is

(40) $$P\left(x_{f_{1}}^{*}\in X_{f_{2}}^{r}\right)=\frac{r}{|X_{O(1)}|}\gg \frac{r}{\left|X\right|}$$

because both $x_{f_{1}}^{*}\in X_{O(1)}$ and $X_{f_{2}}^{r}\subset X_{O(1)}$ due to being simple. Overall, this section has shown that when optima are simple, as a null model expectation, it is exponentially more likely that an optimum solution to one simple combinatorial problem is also highly optimal (or extremely suboptimal) for another arbitrary simple function.

In refs. [61,62,63], there is more exploration of the connection between Kolmogorov complexity and coincidences.

## 7. Discussion

We have studied some connections between Kolmogorov complexity and optima in combinatorial optimization problems. Our main findings are to explore four cases of pairings, namely when the optimization problem is simple or complex, and when the optimum/optima is/are simple or complex. For each case, we showed examples to illustrate. Our second main result was to introduce a sampling method for solving optimization problems, namely by sampling potential solutions according to their algorithmic probability. We showed that this method will exponentially reduce the expected waiting time when the optimum is simple, and yet only lower the expected waiting time for complex optimization problems by a constant factor. Thus, this one method could be used for a variety of problems of differing complexity, and even if the complexity of the optimum is not known. Our third main result was to show that given a geometry/configuration is optimal for one simple objective function, it is therefore more likely to be close to optimal for another simple objective function, as compared to a null expectation based on random functions. This is a rather surprising result, given that we typically conceive of exponentially large search spaces. Importantly, we discussed and highlighted that ‘simple’ in terms of low Kolmogorov complexity does not necessarily imply that the geometry/configuration will be simple in the sense of regular, symmetric, or repeating patterns, but, on the other hand, there are reasons to think that these meanings may, in fact, coincide.

Our results may perhaps help to understand the examples of discrete geometries given in the Introduction (lattice proteins and networks) which simultaneously are simple, regular, and also optimal for a number of different objective functions. We leave it to further work to explore this application of our results in more detail. Similarly, our results may help to explain in a general setting the results of Cohn and Kumar [64], who found that symmetrical configurations can often be built using very simple potential functions, and perhaps also the statement of Bormashenko [65] that the symmetry of biological structures follows the symmetry of media in which the structure is functioning.

In ref. [33] (but also see [42]), it was argued that some of the symmetry in biology can be explained by the fact that simpler shapes are easier to generate via efficient genomic programs in DNA, which is a kind of ‘internal’ argument for simplicity. Here, we have shown that symmetry and simplicity may arise from a kind of ‘external’ argument, that of the simplicity of the external constraints.

Our work is also closely related to earlier studies of the Kolmogorov complexity of a set and computational complexity [66], and also to optimization problems where it was shown that expected complexity of the set is a good measure of problem difficulty [67].

The work here has focused on discrete geometries, configurations, or sequences, such as binary strings, discrete graphs, branching patters, and lattice shapes. However, continuous curves, functions and shapes can be discretised often quite easily [31], and because of this, the arguments presented here apply to a wide array of shapes and geometries.

The size of an object is relevant when applying concepts from Kolmogorov complexity. It is well known from analytical arguments [18] that for small data objects such as short binary strings, some results from AIT may not apply. This is because many results in AIT involve O(1) terms, which formally become negligible only in the limit of large complexity values. Hence, for very small binary strings, AIT results may not be manifested [43], or at least it is not possible to prove that they will be manifested. Additionally, in numerical work examining simplicity bias in RNA structures, it was reported that the bias only becomes apparent for larger lengths [31]. In this study, we have mainly studied optimization in generic terms where the configuration/geometry/object sizes are parameterized by *n*. Hence, our analytical results do not suffer from the potential limitations of using short strings. Having said that, it is noteworthy and interesting to highlight that in potential applications of our results to ‘small’ configurations/geometries/objects in the real world, the small sizes may ‘wash out’ any manifestations of AIT predictions. Hence, we would expect our results and connections of complexity to optimization to be more likely to manifest in larger data objects. This size-dependence presents a limitation of our work for real-world applications.

Looking at individual systems, we may be able to use system-specific theory to directly infer that some optimal shape for one objective function is linked to optimal shapes for some other objective function, e.g., employing thermodynamics to understand why high genetic mutational robustness and thermal stability are linked in natural proteins [68]. Importantly, such examples do not undermine our work here, in which we have attempted to show a general theory linking optima, low complexity, and coinciding optima. Whether or not particular cases can or cannot be understood with existing system-specific theory, does not affect our arguments.

Overall, our results show an interesting and important link between combinatorial optimization and Kolmogorov complexity, which we suggest should be further explored and developed in future work.

## Data Availability

There are no related data for this study.

## References

[1] Hilderbrandt S., Tromba A. (1996). The Parsimonious Universe: Shape and Form in the Natural World.

[2] Mezard M., Montanari A. (2009). Information, Physics, and Computation.

[3] Cohn H. (2010). Order and disorder in energy minimization. Proceedings of the International Congress of Mathematicians 2010 (ICM 2010), Hyderabad, India, 19–27 August 2010.

[4] Cohn H., Kumar A. (2007). Universally optimal distribution of points on spheres. J. Am. Math. Soc..

[5] Li H., Helling R., Tang C., Wingreen N. (1996). Emergence of preferred structures in a simple model of protein folding. Science.

[6] Mélin R., Li H., Wingreen N., Tang C. (1999). Designability, thermodynamic stability, and dynamics in protein folding: A lattice model study. J. Chem. Phys..

[7] Dias C., Grant M. (2006). Designable structures are easy to unfold. Phys. Rev. E.

[8] Greenbury S.F., Schaper S., Ahnert S.E., Louis A.A. (2016). Genetic correlations greatly increase mutational robustness and can both reduce and enhance evolvability. PLoS Comput. Biol..

[9] Nelson E., Teneyck L., Onuchic J. (1997). Symmetry and kinetic optimization of proteinlike heteropolymers. Phys. Rev. Lett..

[10] Valverde S., Cancho R., Sole R. (2002). Scale-free networks from optimal design. EPL (Europhys. Lett.).

[11] Cohen R., Havlin S. (2003). Scale-free networks are ultrasmall. Phys. Rev. Lett..

[12] Solomonoff R.J. (1960). A Preliminary Report on a General Theory of Inductive Inference (Revision of Report V-131). Contract AF.

[13] Kolmogorov A. (1965). Three approaches to the quantitative definition of information. Probl. Inf. Transm..

[14] Chaitin G.J. (1975). A theory of program size formally identical to information theory. J. ACM.

[15] Lloyd S. (2001). Measures of complexity: A nonexhaustive list. IEEE Control Syst. Mag..

[16] Mitchell M. (2009). Complexity: A Guided Tour.

[17] Turing A.M. (1936). On computable numbers, with an application to the Entscheidungsproblem. J. Math..

[18] Li M., Vitanyi P. (2019). An Introduction to Kolmogorov Complexity and Its Applications.

[19] Delahaye J., Zenil H. (2012). Numerical Evaluation of Algorithmic Complexity for Short Strings: A Glance into the Innermost Structure of Algorithmic Randomness. Appl. Math. Comput..

[20] Leyva-Acosta Z., Acuña Yeomans E., Hernandez-Quiroz F. (2024). An additively optimal interpreter for approximating Kolmogorov prefix complexity. Entropy.

[21] Li Z., Huang C., Wang X., Hu H., Wyeth C., Bu D., Yu Q., Gao W., Liu X., Li M. (2025). Lossless data compression by large models. Nat. Mach. Intell..

[22] Grunwald P., Vitányi P. (2004). Shannon information and Kolmogorov complexity. arXiv.

[23] Bennett C. (1982). The thermodynamics of computation—A review. Int. J. Theor. Phys..

[24] Kolchinsky A., Wolpert D.H. (2020). Thermodynamic costs of Turing machines. Phys. Rev. Res..

[25] Zurek W. (1989). Algorithmic randomness and physical entropy. Phys. Rev. A.

[26] Ebtekar A., Hutter M. (2025). Foundations of Algorithmic Thermodynamics. Phys. Rev. E.

[27] Ebtekar A., Hutter M. (2024). Modeling the Arrows of Time with Causal Multibaker Maps. Entropy.

[28] Avinery R., Kornreich M., Beck R. (2019). Universal and accessible entropy estimation using a compression algorithm. Phys. Rev. Lett..

[29] Martiniani S., Chaikin P.M., Levine D. (2019). Quantifying hidden order out of equilibrium. Phys. Rev. X.

[30] Vitányi P.M. (2013). Similarity and denoising. Philos. Trans. R. Soc. A Math. Phys. Eng. Sci..

[31] Dingle K., Camargo C.Q., Louis A.A. (2018). Input–output maps are strongly biased towards simple outputs. Nat. Commun..

[32] Cilibrasi R., Vitányi P.M.B. (2005). Clustering by compression. IEEE Trans. Inf. Theory.

[33] Johnston I.G., Dingle K., Greenbury S.F., Camargo C.Q., Doye J.P., Ahnert S.E., Louis A.A. (2022). Symmetry and simplicity spontaneously emerge from the algorithmic nature of evolution. Proc. Natl. Acad. Sci. USA.

[34] Calude C. (2002). Information and Randomness: An Algorithmic Perspective.

[35] Gács P. (1988). Lecture Notes on Descriptional Complexity and Randomness.

[36] Shen A., Uspensky V.A., Vereshchagin N. (2022). Kolmogorov Complexity and Algorithmic Randomness.

[37] Hutter M. The Loss Rank Principle for Model Selection. Proceedings of the 20th Annual Conference on Learning Theory (COLT’07).

[38] Borges J.L. (1998). The Library of Babel; Collected Fictions.

[39] Dingle K., Schaper S., Louis A.A. (2015). The structure of the genotype–phenotype map strongly constrains the evolution of non-coding RNA. Interface Focus.

[40] Lorenz R., Bernhart S.H., Zu Siederdissen C.H., Tafer H., Flamm C., Stadler P.F., Hofacker I.L. (2011). ViennaRNA Package 2.0. Algorithms Mol. Biol..

[41] Wolfram S. (1985). Undecidability and intractability in theoretical physics. Phys. Rev. Lett..

[42] Wolfram S. (2002). A New Kind of Science.

[43] Vitányi P. (2020). How incomputable is Kolmogorov complexity?. Entropy.

[44] Sethna J. (2006). Statistical Mechanics: Entropy, Order Parameters, and Complexity.

[45] Levin L. (1974). Laws of information conservation (nongrowth) and aspects of the foundation of probability theory. Probl. Peredachi Informatsii.

[46] Hutter M., Legg S., Vitanyi P.M. (2007). Algorithmic probability. Scholarpedia.

[47] Dingle K., Hagolani P., Zimm R., Umar M., O’Sullivan S., Louis A. (2025). Bounding phenotype transition probabilities via conditional complexity. J. R. Soc. Interface.

[48] Valle-Perez G., Camargo C.Q., Louis A.A. (2018). Deep learning generalizes because the parameter-function map is biased towards simple functions. arXiv.

[49] Mingard C., Rees H., Valle-Pérez G., Louis A.A. (2025). Deep neural networks have an inbuilt Occam’s razor. Nat. Commun..

[50] Dingle K., Batlle P., Owhadi H. (2023). Multiclass classification utilising an estimated algorithmic probability prior. Phys. D Nonlinear Phenom..

[51] Hu T., Banzhaf W., Ochoa G. How Neutrality Shapes Evolution: Simplicity Bias and Search. Proceedings of the Genetic and Evolutionary Computation Conference.

[52] Dingle K., Kamal R., Hamzi B. (2023). A note on a priori forecasting and simplicity bias in time series. Phys. A Stat. Mech. Its Appl..

[53] Dingle K., Alaskandarani M., Hamzi B., Louis A.A. (2024). Exploring simplicity bias in 1d dynamical systems. Entropy.

[54] Hamzi B., Dingle K. (2024). Simplicity bias, algorithmic probability, and the random logistic map. Phys. D Nonlinear Phenom..

[55] Ma Y.A., Chen Y., Jin C., Flammarion N., Jordan M.I. (2019). Sampling can be faster than optimization. Proc. Natl. Acad. Sci. USA.

[56] Mingard C., Valle-Pérez G., Skalse J., Louis A.A. (2021). Is SGD a Bayesian sampler? Well, almost. J. Mach. Learn. Res..

[57] Schmidhuber J. The Speed Prior: A New Simplicity Measure Yielding Near-Optimal Computable Predictions. Proceedings of the 15th Conference on Computational Learning Theory (COLT’02).

[58] Grau-Moya J., Genewein T., Hutter M., Orseau L., Deletang G., Catt E., Ruoss A., Wenliang L.K., Mattern C., Aitchison M. Learning Universal Predictors. Proceedings of the 41st International Conference on Machine Learning.

[59] Orseau L., Hutter M., Lelis L.H. Levin Tree Search with Context Models. Proceedings of the 32nd International Joint Conference on Artificial Intelligence (IJCAI’23).

[60] Alaskandarani M., Dingle K. (2023). Low complexity, low probability patterns and consequences for algorithmic probability applications. Complexity.

[61] Kreinovich V. (1999). Coincidences are not accidental: A theorem. Cybern. Syst..

[62] Dessalles J.L.J.L. (2011). Coincidences and the encounter problem: A formal account. arXiv.

[63] Yanofsky N.S. (2019). Kolmogorov Complexity and Our Search for Meaning: What Math Can Teach Us about Finding Order in Our Chaotic Lives. The Best Writing on Mathematics 2019.

[64] Cohn H., Kumar A. (2009). Algorithmic design of self-assembling structures. Proc. Natl. Acad. Sci. USA.

[65] Bormashenko E. (2022). Fibonacci sequences, symmetry and order in biological patterns, their sources, information origin and the Landauer principle. Biophysica.

[66] Borenstein Y., Poli R. (2006). Kolmogorov complexity, optimization and hardness. Proceedings of the 2006 IEEE International Conference on Evolutionary Computation.

[67] Borenstein Y., Poli R. (2006). Information perspective of optimization. Proceedings of the International Conference on Parallel Problem Solving from Nature.

[68] Bloom J.D., Silberg J.J., Wilke C.O., Drummond D.A., Adami C., Arnold F.H. (2005). Thermodynamic prediction of protein neutrality. Proc. Natl. Acad. Sci. USA.

